# Computed tomography–based COVID–19 triage through a deep neural network using mask–weighted global average pooling

**DOI:** 10.3389/fcimb.2023.1116285

**Published:** 2023-03-03

**Authors:** Hong-Tao Zhang, Ze-Yu Sun, Juan Zhou, Shen Gao, Jing-Hui Dong, Yuan Liu, Xu Bai, Jin-Lin Ma, Ming Li, Guang Li, Jian-Ming Cai, Fu-Geng Sheng

**Affiliations:** ^1^ Department of Radiology, the Fifth Medical Center of Chinese PLA General Hospital, Beijing, China; ^2^ Algorithm Center, Keya Medical Technology Co., Ltd, Shenzhen, China

**Keywords:** coronavirus disease 2019 (COVID-19), computed tomography (CT), deep learning, global average pooling (GAP), artificial intelligence

## Abstract

**Background:**

There is an urgent need to find an effective and accurate method for triaging coronavirus disease 2019 (COVID-19) patients from millions or billions of people. Therefore, this study aimed to develop a novel deep-learning approach for COVID-19 triage based on chest computed tomography (CT) images, including normal, pneumonia, and COVID-19 cases.

**Methods:**

A total of 2,809 chest CT scans (1,105 COVID-19, 854 normal, and 850 non-3COVID-19 pneumonia cases) were acquired for this study and classified into the training set (n = 2,329) and test set (n = 480). A U-net-based convolutional neural network was used for lung segmentation, and a mask-weighted global average pooling (GAP) method was proposed for the deep neural network to improve the performance of COVID-19 classification between COVID-19 and normal or common pneumonia cases.

**Results:**

The results for lung segmentation reached a dice value of 96.5% on 30 independent CT scans. The performance of the mask-weighted GAP method achieved the COVID-19 triage with a sensitivity of 96.5% and specificity of 87.8% using the testing dataset. The mask-weighted GAP method demonstrated 0.9% and 2% improvements in sensitivity and specificity, respectively, compared with the normal GAP. In addition, fusion images between the CT images and the highlighted area from the deep learning model using the Grad-CAM method, indicating the lesion region detected using the deep learning method, were drawn and could also be confirmed by radiologists.

**Conclusions:**

This study proposed a mask-weighted GAP-based deep learning method and obtained promising results for COVID-19 triage based on chest CT images. Furthermore, it can be considered a convenient tool to assist doctors in diagnosing COVID-19.

## Introduction

At the beginning of 2020, the coronavirus disease 2019 (COVID-19) infection spread rapidly worldwide. The symptoms of COVID-19 infection are similar to common pneumonia such as pneumonia caused by bacteria and influenza viruses, mainly including fever, malaise, dry cough, and sore throat (Guan et al., 2020; Jiang et al., 2020). Early detection and diagnosis of patients with COVID-19 infection can greatly hinder the spread of the disease and alleviate the patient’s symptoms. Therefore it is an urgent need to find an effective and accurate method for diagnosing COVID-19 patients from common pneumonia.

Currently, diagnosing COVID-19 relies largely on reverse transcription-polymerase chain reaction (RT-PCR) testing of samples from the throat (Ai et al., 2020). However, RT-PCR for COVID-19 diagnosis has some limitations: the test is not universally available, turnaround times can be lengthy, and the reported sensitivities vary. Patients with respiratory symptoms who do not have a confirmed diagnosis of COVID-19 may undergo computed tomography (CT) for different indications, including the diagnosis of suspected pneumonia. CT imaging is another critical tool in the initial screening of COVID-19 pneumonia, serves as an alternative or adjunct to RT-PCR diagnosis, and plays a vital role in early detection, observation, and evaluation of the disease (Bao et al., 2020; Ye et al., 2020; Garg et al., 2022). However, chest CT images usually consist of approximately 100 slices, and it is very time-consuming for radiologists to check whether they are COVID-19 images. With the rapid spread of COVID-19 virus and the large increase in CT data, the development of computer-aided detection system with artificial intelligence to assist radiologists in the diagnosis of COVID-19 patients has become an urgent and necessary task.

Therefore, this study aims to propose a mask-weighted global average pooling (GAP)-based deep learning method for COVID-19 triage based on chest CT images. COVID-19 triage is a slightly different problem compared to common classification problems (Jaipurkar et al., 2018; Wodzinski et al., 2019; Prakash et al., 2022; Ker et al., 2019). Conventional CNNs perform convolution operations in the lower layers of the network and concatenate the last convolution layer’s feature map to the fully connected layer, followed by a softmax logistic regression layer, for classification. This structure bridges the convolutional structure with traditional neural network classifiers. It treats convolutional layers as feature extractors, and the resulting feature is classified traditionally. However, fully connected layers have many more parameters, and GAP layers were proposed (Li et al., 2022; Bao et al., 2023). General GAP calculates the mean value of the entire feature image. The fully connected layer maps all pixels as the input for the classifier, and the GAP maps the entire image as one pixel as the input for the classifier. Therefore, the number of parameters and the model complexity are significantly reduced.

In medical images, the pixel information is much more associated with the concrete clinical structure. Furthermore, GAP with different weight factors according to the different tissues will be useful in reducing the inference of background noise. Therefore, in the medical image classification task, the entire image is sometimes unrequired, and a specified organ region of the image is sufficient. In the COVID-19 triage particularly, COVID-19-related suspicions are all in the lung region, and clinical information is much more useful for this triage problem.

In this paper, we developed an AI algorithm using a mask–weighted GAP method for COVID-19 triage. The main novelty of this paper are summarized:

1. We developed a computer-aided diagnostic algorithm with a 3D convolutional neural network to achieve the diagnosis of COVID-19 patients from common pneumonia based on chest CT images.
2. A mask-weighted GAP method which used the segmented lung region mask to reduce the non-lung region inference for the COVID-19 classification was proposed to improve the accuracy of the COVID-19 triage.
3. The amount of data used in this study is large, containing a total of 2809 cases, covering several public datasets and a private datasets.

## Related work

During the past two years, many classifications and segmentation deep learning algorithms have been developed to assist radiologists in COVID-19 identification (Harmon et al., 2020; Islam et al., 2020; Fan et al., 2022; Shaik and Cherukuri, 2022) and severity qualification (Li et al., 2020; Qin et al., 2020).

Md. Islam (Islam et al., 2020) proposed a deep learning-based system combining a convolutional neural network (CNN) and long short-term memory (LSTM) networks to detect COVID-19 automatically based on radiographs. In the proposed system, CNN was used for feature extraction, and LSTM was used to classify COVID-19 based on these features. This can help doctors diagnose and treat COVID-19 patients easily. Harmon S A (Harmon et al., 2020) developed and evaluated an AI algorithm for the detection of COVID-19 on chest CT using data from a globally diverse, multi-institution datasets. Fan X (Fan et al., 2022) built a parallel bi-branch model (Trans-CNN Net) based on Transformer module and CNN module is proposed by making full use of the local feature extraction capability of CNN and the global feature extraction advantage of Transformer. Bosowski et al. (Bosowski et al., 2021) introduced deep ensembles that benefit from a wide range of architectural advances, alongside a new fusing approach to deliver accurate predictions of COVID-19 cases on a number of datasets of chest X-ray images.

Li et al. (Li et al., 2020) developed a fully automated artificial intelligence system to quantitatively assess the disease severity and progression of COVID-19 using thick-section chest CT images. Le Qin (Qin et al., 2020) developed a predictive model and scoring system to enhance the diagnostic efficiency for COVID-19, and CT features and scores were evaluated at the lung segment level according to the lesion position, attenuation, and form. In most of these studies, the disease severity and progression of COVID-19 have been assessed.

These methods above applied AI techniques to the detection and evaluation of COVID-19 pneumonia based on medical imaging data in different modalities and achieved better performance. However, there are some shortcomings in these methods, either some studies use a small amount of data from a single source, which makes it difficult to verify the generalization ability of the model, or some studies use non-CT data, such as X-ray images, which have low sensitivity in clinical applications. Inspired by the above-mentioned researches, this study proposed a mask–weighted global average pooling–based deep learning method for COVID–19 triage based on chest CT images.

## Material and methods

### Patients

This study included two types of datasets: public and private. The public dataset consisted of two different data sources: COVID-19 lung CT lesion segmentation challenge (An et al., 2020; Roth et al., 2022) (https://covid-segmentation.grand-challenge.org/Data/) and MosMeddata (Morozov et al., 2020) (https://www.kaggle.com/datasets/andrewmvd/mosmed-covid19-ct-scans). The first public dataset contained images of 249 COVID-19 patients, while the second contained images of 856 COVID-19 patients and 254 non-pneumonia patients (n = 1,110). The private dataset was acquired from our Hospital, which contained images of 850 pneumonia and 600 normal patients.

These data were mixed, and a total of 2,809 scans were used in this study, including 854 normal, 850 common pneumonia, and 1,105 COVID-19 cases. For each category, 15%-20% of the scans were randomly selected as the test set and the remaining as the training set. To verify the performance of the COVID-19 triage, we made the test set have roughly the same number of CT scans between COVID-19 and non-COVID-19 patients. Information on these datasets is presented in **Table 1**.

**Table 1:** Dataset information.

	Non-pneumonia	CAP	COVID-19
Public Dataset A	0	0	249
Public Dataset B	254	0	856
Private Dataset	600	850	0
Total number	854	850	1,105
Training Set	726	724	879
Test Set	128	126	226

### Workflow

**Figure 1:**
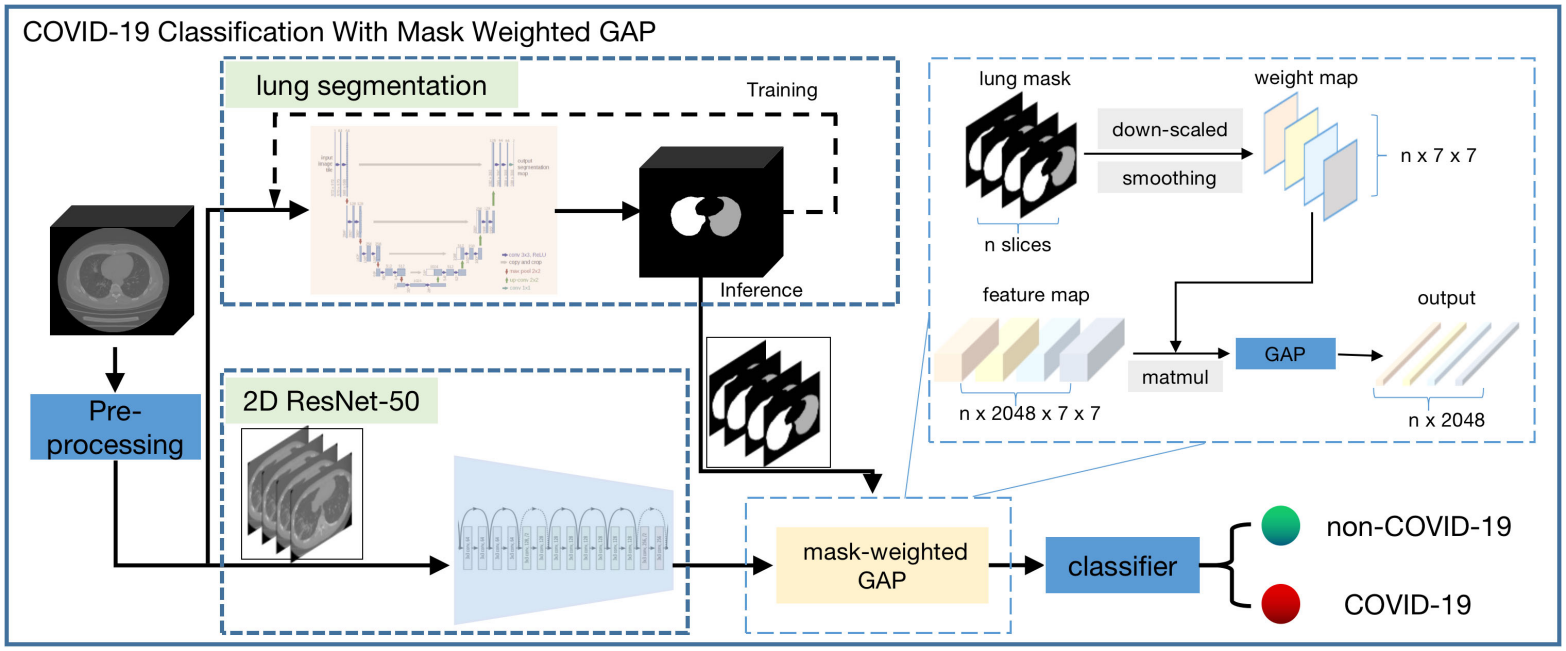
Workflow chart of the whole COVID-19 triage algorithm. There are the following modules: pre-process, lung segmentation, and classification. A 3d-Unet model was used for lung segmentation and the input of this module is the isotropic volume after pre-process module. The cropped isotropic image by the lung mask region is the input of classification module. Before the classifier, a weight-mask GAP is applied to the feature maps extracted by resnet-50 model.

The workflow chart of the COVID-19 triage algorithm is illustrated in **Figure 1**. First, pre–processing was performed on all the data. Second, segmentation of the lung region and cutting out of the lung region mask and CT image were performed. Third, according to the lung region mask and CT image, a deep neural network was used to perform the COVID-19 classification.

### CT image pre-processing

Because the data had different spacings and sizes, the first pre–processing step was image resampling to obtain the isotropic volume, and the pixel spacings between the x, y, and z directions were the same. Subsequently, to reduce the variation between different datasets, we calculated each scan’s mean and standard deviation and used Z-score normalization to normalize the images.

### Lung segmentation

Lung segmentation was based on the CNN method using the U-Net model (Kalpana et al., 2022; Papetti et al., 2022). There are two paths in the model network: the left path, which encodes the image features, and the right path, which decodes the features and localizes the target tissue. The left-contracting path follows the typical architecture of a convolutional network. It consists of the repeated application of two 3 × 3 × 3 convolutions, each followed by a rectified linear unit (ReLU) and a 2 × 2 × 2 max pooling operation with stride 2 for downsampling. Every step in the expansive path consists of an up–sampling of the feature map, followed by a 2 × 2 × 2 convolution that halves the number of feature channels, concatenation with the correspondingly cropped feature map from the contracting path, and two 3 × 3 × 3 convolutions, each followed by a ReLU. Herein, the input of our three–dimensional (3D) U-net model was the output of the pre-processing step, which was the resampled isotropic volume with a size of 128 × 128 × 128. The output of the 3D U-net model was the segmentation mask of the lung region.

The training dataset for lung segmentation was an open dataset named LUNA16 (Armato et al., 2011), which can be accessed on the LUNA16 website. LUNA16 has 888 volumes of lung data, with a slice thickness greater than 2.5 mm. Furthermore, lung segmentation is sufficient for COVID-19 classification, considering the application scenario and cost of manual labeling. Therefore, we randomly selected 300 volumes of data for training and 30 for validation. The same pre–processing method was applied to these data to reduce both the graphics processing unit (GPU) memory limitation and training time.

### COVID-19 classification using mask-weighted GAP

The COVID-19 classification method was based on the residual network (ResNet) (Kibriya and Amin, 2022; Malik et al., 2022; Suganya and Kalpana, 2023), which received the state-of-the-art performance award at the ImageNet Large Scale Visual Recognition Challenge (ILSVRC) 2015 for classification, localization, detection, Common Objects in Context (COCO) detection, and segmentation tasks.

A conventional ResNet was used for one-image classification. However, our input was one series of images, and these 3D CT images can be used in two ways. One method used 3D convolution and modifies the original ResNet to 3D ResNet, and the other used two–dimensional (2D) convolution for the series of images and combines several 2D output features. Herein, we adopted the second method, considering the sparse information on suspected COVID-19 and the large memory usage of 3D convolution. In our classification method, the input CT image and mask were first resized to 224 × 224, and the ResNet model of one image outputted feature maps with a size of 2048 × 7 × 7. Then, a weight-mask GAP method was applied to the feature maps before the classifier layer. Inspired by the idea of attention, we increased the weight factors of lung regions closely related to the COVID-19 classification during GAP operation, which was used to improve the sensitivity of lung regional lesions and reduce the interference of background noise. The mask-weighted GAP calculation is shown in Equation (1).


(1) $$I_{featureweightedGAP}=\sum_{i=0}^{i=7}\sum_{j=0}^{j=7}I_{smoothedmask(i,j)}\times \;I_{feature(i,j)}$$

I_feature_ is the output feature image from the last convolution layer of the ResNet50. I_smoothedmask_ is the lung mask region, downscaled to 7 × 7, followed by Gaussian smoothing; the last normalized summation of all pixel values was 1. The downscale operation made the lung mask the same size as the final feature map, and the smoothing operation made the weighting factor vary smoothly considering the spatial relationship. The conventional GAP was used to obtain the average value of I_feature_; however, the mask–weighted GAP combined the different weights from I_smoothedmask_ and I_feature_ to obtain the final value of I_featureweightedGAP_. If the weighted mask values were all the same, it would be equal to the conventional GAP; particularly, the weighting value of the lung region area would be larger than that of the non–lung region area to guarantee that the feature focused on the lung region containing the suspected COVID-19.

In the COVID-19 classification method, the model’s input was CT image and lung region masks according to the results of the lung segmentation model. The model’s output was the probability of each case being predicted as COVID-19.

### Statistical analysis

We compared the classification performance using several metrics such as accuracy, sensitivity, specificity, F1 score, and area under the curve (AUC) (Lu et al., 2023).

The accuracy of a test is its ability to differentiate between positive and negative cases correctly. The sensitivity of a test is its ability to identify positive cases correctly. The specificity of a test is its ability to identify negative cases correctly. The F1 score is the weighted average of precision and recall.


(2) $$Accuracy=\frac{TP+TN}{TP+TN+FP+FN}$$

(3) $$Sensitivity=\frac{TP}{TP+FN}$$

(4) $$Specificity=\frac{TN}{TN+FP}$$

(5) $$F1score=\frac{2TP}{2TP+FN+FP}$$

True positive (TP) is the number of cases correctly predicted as positive, true negative (TN) is the number of cases correctly predicted as negative, false positive (FP) is the number of cases incorrectly predicted as positive, and false negative (FN) is the number of cases incorrectly predicted as negative.

The AUC is an index used to measure the performance of a classifier. The AUC provides a method to measure the accuracy of a diagnostic test. The larger the area, the more accurate the diagnostic test. The AUC of the receiver operating characteristic (ROC) curve can be measured using the following equation (6), where t = (1 - specificity) and ROC (t) is sensitivity.


(6) $$AUC=\int_{0}^{1}ROC(t)dt$$

For the segmentation task, the validation metric is usually the Dice coefficient (DC). The DC measures the spatial overlap between the two segmentation regions. The DC is 2 × area of overlap divided by the total number of pixels in both images. The larger the DC value, the better the segmentation result. Therefore, DC was adopted to measure the lung segmentation performance in this study.

## Results

All experiments in this study were performed on ubuntu 20.04. The model was trained using the deep learning framework Pytorch 1.8.1 and CUDA 11.4. All experiments were run on one NVIDIA DGX Station, with four NVIDIA Tesla V100 DGXS 32GB GPUs and one Intel(R) Xeon(R) CPU E5-2698 CPU. The model was trained using the optimizer Adam with an initial learning rate of 1e-5, and the learning rate was adjusted using warm up and CosineAnnealingLR.

There were two models for training: lung region segmentation and COVID-19 classification models. The total training epoch for lung segmentation and COVID-19 classification were 100 and 60 epochs, and the training time were around 6 and 10 hours respectively. The following subsections describe the qualitative and quantitative results of the two models.

### Lung region segmentation results

**Figure 2:**
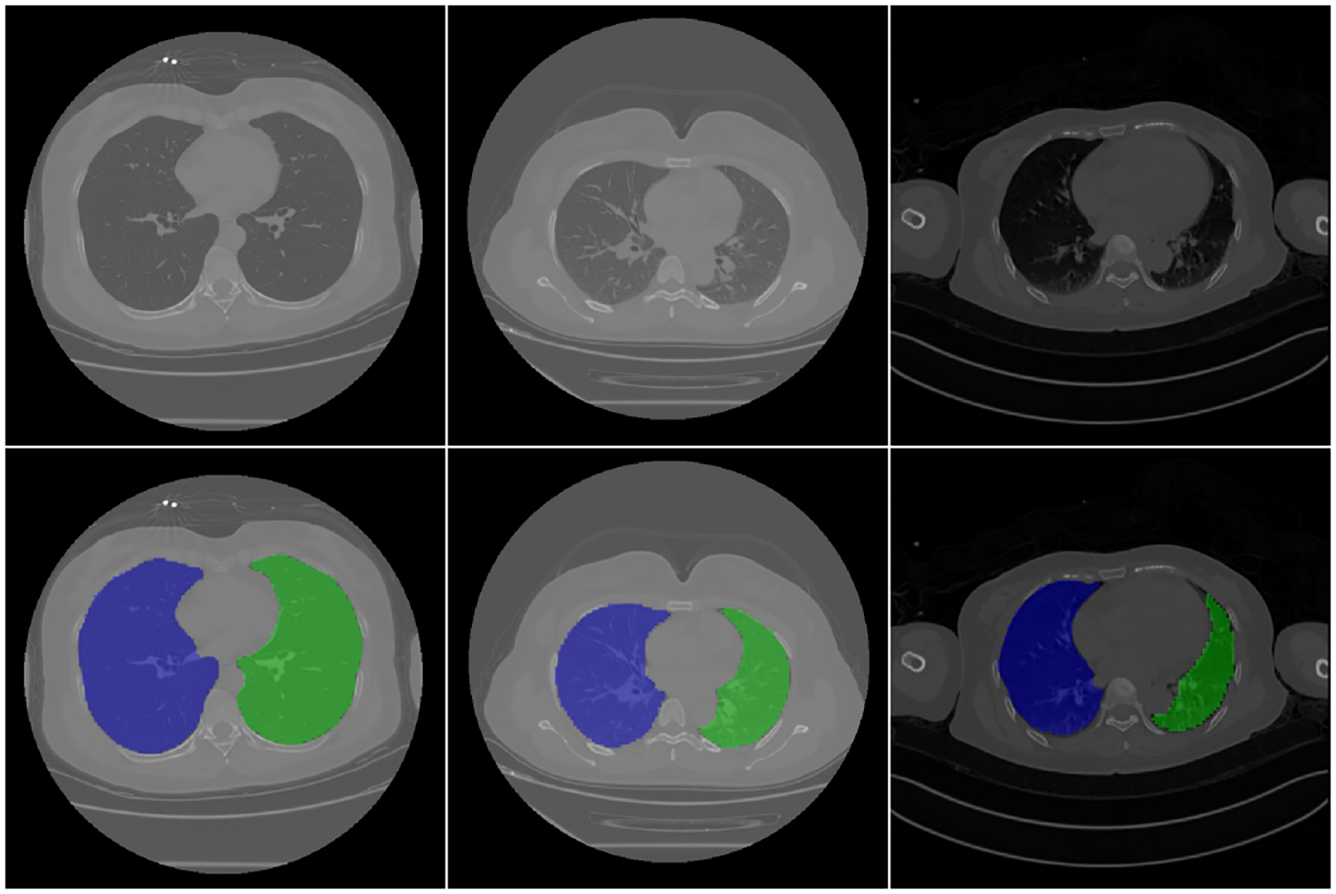
CT images and related mask. First raw are original CT image, second raw are the corresponding segmentation results. In the figure, the background is shown in black, the right lung is shown in blue, and the left lung is shown in green.

The lung region mask was generated using a 3D U-net model. The input was the original CT data, and the output was the mask of the left and right lungs. **Figure 2** shows the different image slices and their related mask. Because lung segmentation is used for COVID-19 classification, being very accurate is unnecessary. Our segmentation result was validated using the DC, which was approximately 96.5% on 30 independent CT scans.

### COVID-19 classification results

The performance of proposed mask-weighted GAP method was evaluated on test set which included 226 COVID-19 CT scans and 254 non-COVID-19 scans. The evaluation metrics were calculated according to the formulations described in the previous section. Our proposed method achieved a sensitivity of 96.5% and a specificity of 87.8%.

For comparison, we also trained a COVID-19 classification models using general GAP and evaluated the performance on the same test set. The results of the quantification metrics comparison between the general GAP and mask-weighted GAP are shown in **Table 2**. Our proposed method achieved a better performance, both the accuracy and F1 score improved by more than 1%. Based on the results, we found that mask-weighted GAP will be useful for medical image classification of suspicions in special clinical organs.

**Table 2:** The classification results of the proposed Mask-weighted GAP and the comparison with normal GAP based on resnet50.

Model (resnet50)	Accuracy	Sensitivity	Specificity	F1 score
Normal GAP	90.4%	95.6%	85.8%	90.4%
Mask-weighted GAP	91.9%	96.5%	87.8%	91.8%

**Figure 3:**
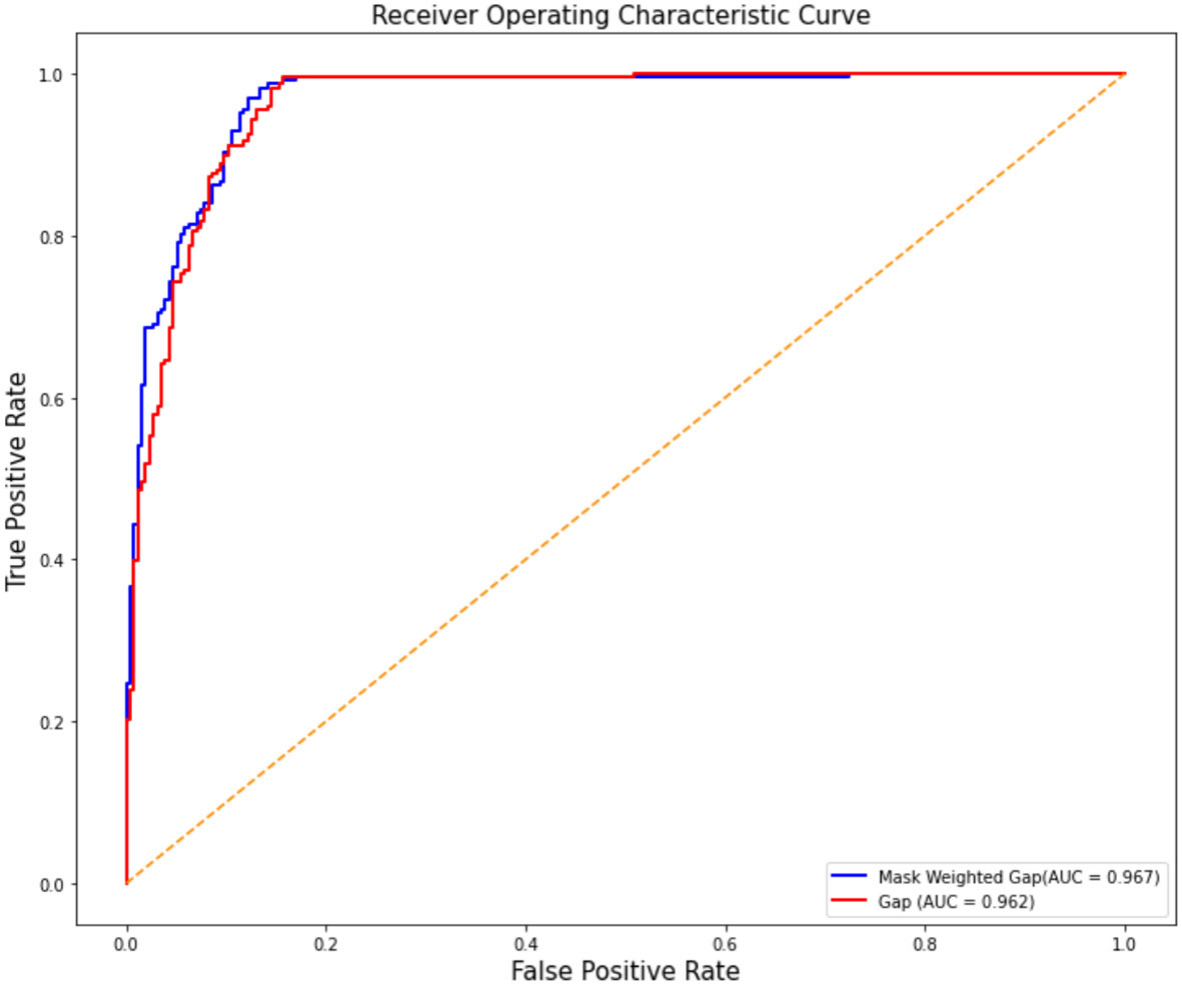
The ROC cure comparison of the two experiment results of resnet50 with GAP and with mask weighted GAP. Vertical axis is false positive rate, while horizontal axis is true positive rate. The blue cure is the result of mask weighted GAP, while the red cure is the result of GAP.

The ROC curve comparison between the experimental results of ResNet50 with GAP and mask-weighted GAP is shown in **Figure 3**. The mask-weighted GAP obtained an AUC value of 0.967 for COVID-19 classification, whereas the general GAP method obtained an AUC value of 0.962. Therefore, the mask-weighted GAP will be more useful for medical image classification of suspicions in special clinical organs because of the attention to regions containing these suspicions.

### Visualization check of COVID-19 triage

**Figure 4:**
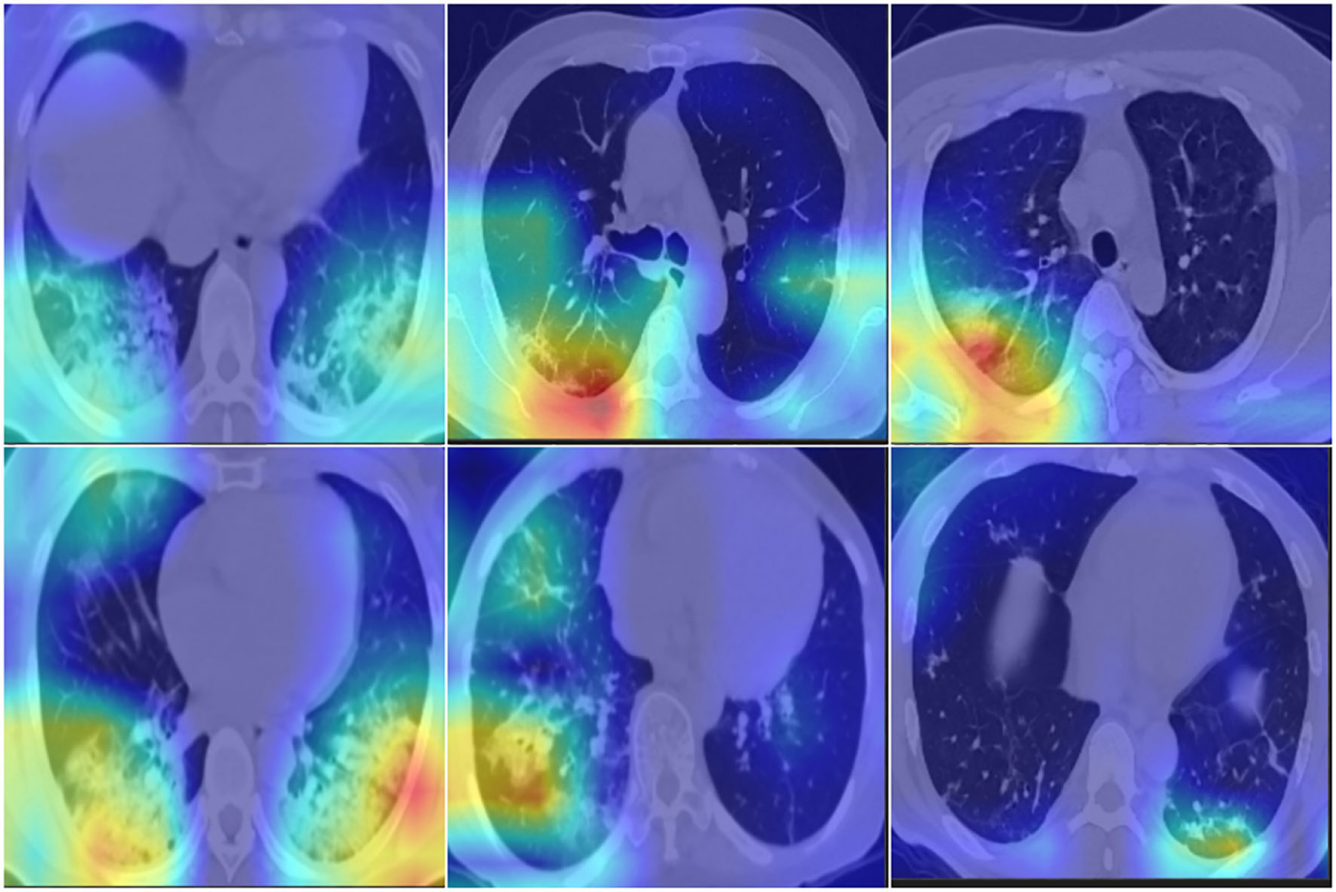
Fusion images with Grad-CAM, which indicate the import region for classification. First row are normal cases, second row are COVID-19 cases. RGB color indicate the high risk for suspects, light color indicate the normal region.

To confirm the COVID-19 triage result, Grad-CAM (Zhao et al., 2023) was adopted to fuse the key region for the classification decision on the original image. A good visualization method uses heavy colors to highlight the suspected region and light colors to indicate the normal region. The colored region is the most important in deciding whether the current image is a COVID-19 image; red indicates a high probability, while green and blue are the next priority. The fusion image will not include a significantly colored region if the CT image is normal. **Figure 4** shows the suspected COVID-19 region highlighted in red; however, the normal region on the CT image is without a significant color. The fusion color map is useful in confirming the importance of CNN feature regions to distinguish COVID-19 suspects.

## Discussion

In this study, we developed and evaluated a lung mask-weighted GAP-based deep learning method for COVID-19 triage based on chest CT scans. A total of 2,809 scans, including 854 normal, 850 common pneumonia, and 1,105 COVID-19 cases, were used for classification, and 330 data volumes with lung masks were used for lung segmentation. The U-net-based model achieved a DC of approximately 96.5% in the lung segmentation task, and the mask-weighted GAP method achieved an accuracy of 91.9%, sensitivity of 96.5%, specificity of 87.8%, and AUC of 96.7% in the COVID-19 classification task. In addition, a Grad-CAM method was adopted to confirm the COVID-19 triage results.

The lung mask-weighted GAP achieved better performance than the normal GAP classification method. The lung mask-weighted GAP showed a 1% improvement in all metrics. This may be because the lung mask-weighted GAP method focuses the model on the lung region containing the suspected COVID-19, reduces the influence of the background region, and highlights the classification features of tissue regions. In addition, the lung mask-weighted GAP could also improve the sensitivity of suspect feature contribution and specificity by reducing the effects of artifacts in the lung region, such as ground–glass opacity or consolidation features. In addition, according to the ROC curve, our COVID-19 classification model is a high-sensitivity model, which is important in screening COVID-19 patients. In our experiments, the AI algorithm took only 10s to complete the classification of one case, which can reduce the pressure of radiologists and improve the efficiency of diagnosis.

This study also had several limitations. First, we only performed COVID-19 diagnosis based on chest CT scans using the deep learning method. While we developed an algorithm to detect the infection lesion, this study did not report a quantitative analysis of the infection lesion. Second, respiration and heart motion due to motion artifacts may reduce the accuracy of the deep learning method. However, this study excluded several severe motion artifact cases. In the future, the training data should include motion artifact cases for both COVID-19 and normal scans. Finally, our study data included COVID-19, pneumonia, and normal cases; therefore, the diagnosis of these three cases should be developed using a deep learning method in future work.

In this study, we have verified the effect of mask-weighted on GAP. In the future, we will apply the mask-weighted method to more methods such as attention model (Rehman et al., 2023) and transformer model (Yang et al., 2023), hoping to further improve the performance.

## Conclusions

A lung mask–weighted GAP-based deep learning method was developed to diagnose COVID-19 and non-COVID-19 cases based on chest CT scans. The evaluation results confirmed that this deep learning-based method was feasible.

## Data availability statement

The datasets presented in this study can be found in online repositories. The names of the repository/repositories and accession number(s) can be found in the article/supplementary material.

## Ethics statement

This study has been approved by the appropriate ethics committee and all persons gave their informed consent prior to their inclusion in the study.

## Author contributions

H-TZ and F-GS conceived and designed the study. H-TZ, JZ and SG wrote the paper and analysed data. J-HD, YL, XB collected data and analysed imaging. J-LM and ML analysed imaging. Z-YS and GL provided technical support. J-MC was responsible for revising the content manuscript. All authors contributed to the article and approved the submitted version
